# Dental Implantation Changes the Bone Morphology and Mineral Density of Human Mandibular Condyle: A Pilot Study

**DOI:** 10.3390/jfb17020099

**Published:** 2026-02-18

**Authors:** Ian Segall, Mark Finkelstein, Sonya Kalim, Jinju Kim, Nicholas Jones, Zachary Skabelund, Hong Chen, Hany A. Emam, Lisa Knobloch, Do-Gyoon Kim

**Affiliations:** 1 Division of Orthodontics, College of Dentistry, The Ohio State University, Columbus, OH 43210, USA; isegall12@gmail.com (I.S.); finkelstein.28@buckeyemail.osu.edu (M.F.); jinju@youngki.org (J.K.); jones.7366@buckeyemail.osu.edu (N.J.); zachskabelund@gmail.com (Z.S.); 2 Workman School of Dental Medicine, High Point University, High Point, NC 27262, USA; skalim@highpoint.edu; 3 Division of Oral and Maxillofacial Pathology, College of Dentistry, The Ohio State University, Columbus, OH 43210, USA; chen.13077@osu.edu; 4 Division of Oral and Maxillofacial Surgery and Anesthesiology, College of Dentistry, The Ohio State University, Columbus, OH 43210, USA; emam.4@osu.edu; 5 Division of Restorative and Prosthetic Dentistry, College of Dentistry, The Ohio State University, Columbus, OH 43210, USA; knobloch.3@osu.edu

**Keywords:** dental implantation, temporomandibular joint, mandibular condyle, cone beam computed tomography, bone mineral density

## Abstract

Dental implantation affects masticatory bite and muscle forces. The temporomandibular joint (TMJ) bears a substantial amount of these masticatory forces. Thus, the objective of the current study was to investigate whether dental implantation alters the human mandibular condyle. Among 556 images, 54 and 22 CBCT scans were successfully identified from 27 patients (10 males and 17 females; 54.93 ± 19.46 years) in the control group and 11 patients (3 males and 8 females; 51.32 ± 13.13 years) in the implant group, respectively. In the control group, CBCT images were obtained longitudinally at the time of implantation and after the post-implantation healing period, both prior to crown placement. In the implant group, CBCT images were obtained at the time of crown placement on a single-tooth implant and after the functional loading period following crown placement. Left and right mandibular condyles were digitally isolated from the images. The bone mineral density (BMD) parameters and morphological changes were assessed using frequency plots of BMD and TMJ osteoarthritis (OA) counts, respectively. In the control group, BMD values were not significantly different between the first and second scans. In contrast, the implant group showed a significant decrease in BMD values, along with a marginal increase in TMJ OA counts after the functional loading period. The TMJ OA counts were highest in the anterior regions, followed by the middle and posterior regions. Most regions showed significantly reduced BMD values, except the antero-lateral and antero-central regions. The current findings give an insight that dental implantation may alter the morphology and BMD of human mandibular condyles. The TMJ OA counts increased, while BMD decreased during the functional loading period of more than 3 months following implantation. Masticatory loading associated with the dental implant likely increases the load on the TMJ, which could stimulate new bone formation to balance the load distribution on the mandibular condyle.

## 1. Introduction

Dental implants have been used to replace missing teeth. About 5 million dental implantations are performed annually and continue to rise as the elderly population expands [1,2]. It is projected that the prevalence of dental implantation will increase up to 23% of the US population by 2026 [3]. A natural tooth root is surrounded by the periodontal ligament (PDL) that helps dissipate the dynamic impact loading energy of mastication on teeth [4,5,6]. On the other hand, the masticatory force applied on the dental implant is directly transmitted to the peri-implant bone that is contacted on the implant surface by osseointegration [7,8]. As such, the interfacial bone adjacent to the dental implant sustains greater masticatory loading energy than the alveolar bone adjacent to the PDL.

Numerous clinical studies have observed that the placement of dental implants improves masticatory efficiency by increasing bite forces [9,10,11]. Furthermore, when the dental implant system is subjected to excessive load, marginal bone loss is developed due to high stress concentration [8,12,13,14]. In addition, dental implantation has an effect on facial muscle activity during mastication [15,16]. These substantial bite and muscle forces are transmitted to the temporomandibular joint (TMJ) [17,18,19]. However, a lack of knowledge exists about the effects of dental implantation on the TMJ.

Research Diagnostic Criteria (RDC) were used to diagnose TMJ disorders, regarding the biopsychosocial factors of patients [20]. However, only a few diagnostic methodologies have been established to quantify alterations in the TMJ. Recently, a novel approach was suggested to diagnose TMJ osteoarthritis (TMJ OA), which can quantify the morphological alterations in the mandibular condyle using clinical cone beam computed tomography (CBCT) images of patients [21]. The CBCT-based TMJ OA counts were successfully utilized to quantify the differences of mandibular condyle shapes between male and female groups [22]. Furthermore, the previous study quantified the bone mineral density (BMD) of the mandibular condyle using the CBCT images, providing insights into the biological consequences of bony changes [22]. We hypothesize that CBCT-based BMD and morphological analyses may reflect biological bony changes in the TMJ under additional articulating loads induced due to dental implantation. In the current study, CBCT-based TMJ OA counts and the BMD distribution of the human mandibular condyle were compared at the time of crown placement on a single-tooth implant and after the functional loading period following crown placement. These approaches enabled us to address the objective of the current study, which was to investigate whether dental implantation alters the human mandibular condyle.

## 2. Materials and Methods

### 2.1. Cone Beam Computed Tomography (CBCT)

**Figure 1:**
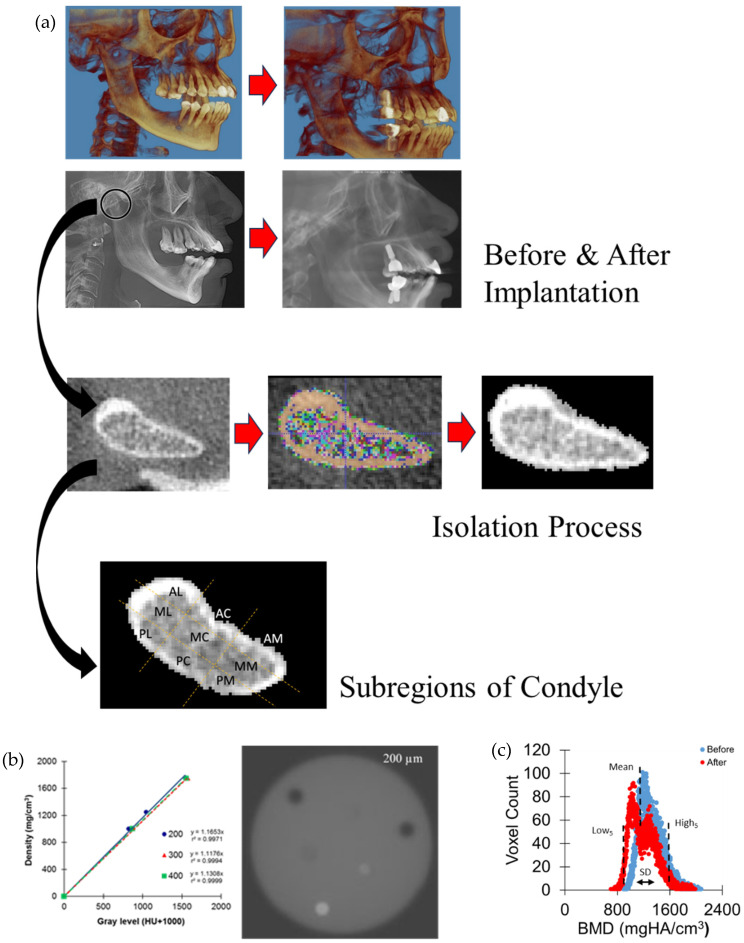
(**a**) Processes for analyzing cone beam computed tomography (CBCT) images and bone mineral density (BMD). Three-dimensional CBCT image of a patient’s mandibular condyle was digitally isolated and divided to subregions of AM (antero-medial), AC (antero-central), AL (antero-lateral), MM (mid-medial), MC (mid-central), ML (mid-lateral), PM (postero-medial), PC (postero-central), and PL (postero-lateral). (**b**) CBCT gray levels at 3 different resolutions (200, 300, and 400 µm) had strong linear relationships with 3 different densities (1000, 1250, and 1750 mgHA/cm^3^) of hydroxyapatite (HA) phantoms, which allowed us to convert the gray level to bone mineral density (BMD) (reprinted with unrestricted permission for non-commercial use [23]). (**c**) BMD parameters in a frequency plot for the mandibular condyle between the time of crown placement on the implant (before) and after the functional loading period following crown placement on the implant (after).

After approval from the Institutional Review Board (IRB) at the Ohio State University (Protocol no. 2011H0128), CBCT images were obtained from the various dental clinics of the College of Dentistry. All patients were scanned using the same CBCT machine (iCAT, Imaging Science International, Hatfield, PA, USA) with identical acquisition parameters routinely used in clinical practice, including a large field of view (FOV), voxel size of 0.3 mm, 120 kV, 5 mA, and a scan time of 8.9 s (Figure 1a).

The sample size was calculated using CBCT-based mean gray values from a previous study that compared pre- and post-treatment outcomes (966.59 ± 106.78 vs. 1060.95 ± 66.01) over 20.05 ± 4.18 months [24]. Based on these data, we determined that a sample size of five CBCT images would provide the minimum number required to achieve statistically significant results (*p* < 0.05) with 95% statistical power using a paired two-sample *t*-test. We used the CBCT values from the previous study because it employed longitudinal scanning with follow-up intervals that fell within the current study’s scanning interval (3 to 33.27 months).

Among 556 images, 54 and 22 CBCT scans were successfully identified from 27 patients (10 males and 17 females; 54.93 ± 19.46 years) in the control group and 11 patients (3 males and 8 females; 51.32 ± 13.13 years) in the implant group, respectively. CBCT images were excluded if they demonstrated the presence of surgical reconstructive hardware, incomplete capture of essential TMJ osseous structures, or absence of posterior occlusion.

In the control group, CBCT images were obtained at the time of implantation and after the post-implantation healing period, both prior to crown placement. Therefore, masticatory loading was not directly applied to the implants during the healing period in control patients.

In the implant group, scans were obtained at the time of crown placement on a single-tooth implant (before) and after the functional loading period following crown placement (after). The interval between scans ranged from 3 to 33.27 months (13.68 ± 6.82 months). The duration of the human bone remodeling cycle has been reported to be approximately 120–200 days [25,26]. Therefore, the BMD distribution observed at the 3-month follow-up likely reflects bone modeling and remodeling during the functional loading period following crown placement.

Articulating loads continuously applied to the TMJ can induce bone modeling and remodeling beyond this 3-month period. For the implant group, patients with full arch prostheses, such as an implant-supported or implant-fixed denture, or with a history of any obvious major head and neck surgical procedures, such as orthognathic surgery, were not considered for this study. The locations of single-tooth implants were randomly distributed in the maxilla and mandible, including 3 in the upper right, 1 in the upper left, 5 in the lower right, 3 in the lower left, 5 in the upper anterior, and 2 in the lower anterior regions.

### 2.2. Temporomandibular Joint Osteoarthritis (TMJ OA) Counts

Right and left mandibular condyles were digitally dissected at 7 mm (23 voxels) down from an apex point of the condylar head in the axial view using imaging software (ImageJ, 1.47v, NIH) (Figure 1a). Twenty-three axial images of the condyle were isolated, ranging from the inferior base of the condyle to the widest portion of the superior aspect of the condyle. These 23 images were then imported into ITK-SNAP software (v3.2, http://www.itksnap.org/pmwiki/pmwiki.php, accessed on 5 December 2025) to segment the mandibular condyle voxels. This program allowed users to change the cross-sectional view of the images to ensure all components of the condyle were included in the analysis. For the implant group, the isolated mandibular condyle image was further divided into 9 sections, including antero-medial (AM), antero-central (AC), antero-lateral (AL), mid-medial (MM), mid-central (MC), mid-lateral (ML), postero-medial (PM), postero-central (PC), and postero-lateral (PL), following the previous study that identified radiographic characteristics related to TMJ OA based on 3D CBCT images [21]. Radiographic characteristics associated with TMJ OA, including flattening, erosions, osteophyte formation, sclerosis, and subchondral cyst formation, were counted by an experienced Oral and Maxillofacial Radiologist (S.K.) using the protocol suggested in the previous study [21]. This study simply aimed to identify any significant osseous changes in the condyle and not necessarily diagnose disease progression.

### 2.3. Bone Mineral Density (BMD) Distribution

The gray level of each voxel was adjusted to a positive value by adding 1000 because the lowest gray level in the image was −1000 for air voxels. This adjustment enables the comparison of absolute positive gray levels between images. The gray levels of each voxel in the isolated mandibular condyle were proportionally converted to bone mineral density (BMD) to produce a BMD frequency plot (Figure 1b) as established in previous studies [22,23,24,25,26]. It was found that there were strong positive relationships between gray levels and BMDs with r^2^ > 0.99. The mean BMD value (Mean) was computed by dividing the sum of BMD values by the total voxel counts in the mandibular condyle. Standard deviation (SD) of the frequency plot represents the heterogeneity of the BMD distribution (Figure 1c). Fifth and ninety-fifth percentiles of the frequency plot were also obtained (Low_5_ and High_5_, respectively).

### 2.4. Statistical Analysis

Reliability tests for TMJ OA counts were examined by an intra-class correlation coefficient (ICC) using TMJOA counts assessed by the Oral and Maxillofacial Radiologist (S.K.) from 10 randomly selected condyles. A Shapiro–Wilk test was performed to confirm the normality of TMJ OA counts. If the data distribution was not normal, the Wilcoxon test was used to compare the TMJ OA counts in the first and second images. A paired *t*-test was used to compare BMD parameters between the left and right sides of the mandibular condyle and its subregions in the first and second CBCT images. A mixed repeated measures analysis of variance (RMANOVA) with an individual patient as a random factor was performed to compare BMD Mean and Low_5_ values between subregions. Statistical significance was set at *p* < 0.05.

## 3. Results

**Figure 2:**
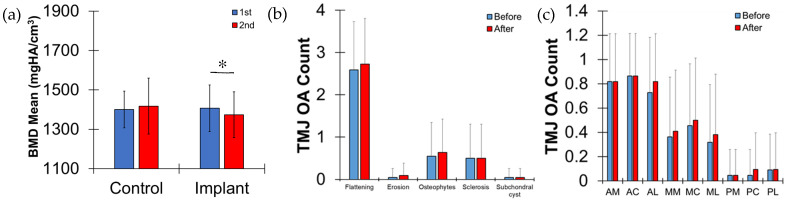
(**a**) Comparison of bone mineral density (BMD) mean values for the control and implant groups between the first and second scans. (**b**) Temporomandibular joint osteoarthritis (TMJ OA) counts for radiographic characteristics (flattening, erosions, osteophyte formation, sclerosis, and subchondral cyst formation), and (**c**) paired comparisons across subregions (antero-medial (AM), antero-central (AC), antero-lateral (AL), mid-medial (MM), mid-central (MC), mid-lateral (ML), postero-medial (PM), postero-central (PC), and postero-lateral (PL)). Flattening dominated and the three anterior regions (AM, AC, and AL) had a significantly higher prevalence of TMJ OA counts than other regions (*p* < 0.05). No significant differences were found between the time of crown placement on the implant (before) and after the functional loading period following crown placement on the implant (after) (*p* > 0.16). *; 0.024.

The values of BMD Mean were not significantly different between the first and the second scans for the control group (*p* = 0.23), but the implant group showed a significant decrease in BMD values (*p* < 0.025) (Figure 2a). Therefore, the current study focused on analyzing parameters only for the implant group.

No severe TMJ disorder was observed for either the left or right sides of the mandibular condyle. Nine subregions were successfully isolated using individual 3D CBCT images. The ICC for the TMJ OA count was 0.92 (*p* < 0.001). Flattening was the most frequently observed (Figure 2b). During the functional loading period following crown placement on the implant, the TMJ OA count marginally increased (*p* = 0.058), while significantly decreasing the values of the BMD Mean and Low_5_ of the condyle (*p* < 0.024) (Table 1). The values of BMD Mean and Low_5_ marginally decreased on the right side of the condyle (*p* < 0.06), while those on the left side of condyle were not significantly different (*p* > 0.112). The right side of the condyle had significantly lower values of BMD Mean and Low_5_ before the functional loading on the implant (*p* < 0.04) and BMD Low_5_ after loading (*p* = 0.016) than the left side of the condyle. All other parameters measured on the condyle were not significantly different between before and after the functional loading on the implant (*p* > 0.068).

**Figure 3:**
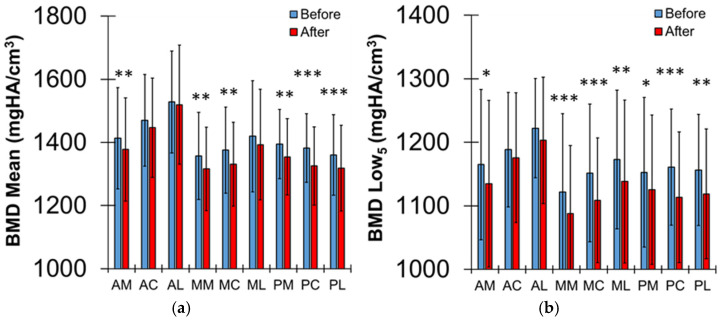
Paired comparisons of bone mineral density (BMD) (**a**) Mean and (**b**) Low_5_ values across subregions (antero-medial (AM), antero-central (AC), antero-lateral (AL), mid-medial (MM), mid-central (MC), mid-lateral (ML), postero-medial (PM), postero-central (PC), and postero-lateral (PL)). BMDs in mandibular condyle regions decreased under functional loading after implantation. *; *p* < 0.05, **; *p* < 0.03, ***; *p* < 0.01.

The TMJ OA counts were highest at the anterior regions, followed by the middle and posterior regions (Figure 2c). The AL and AC regions had significantly higher values of BMD Mean than other regions (*p* < 0.02) and the AC region had significantly higher value of BMD Low_5_ than other regions (*p* < 0.011), except the AL (*p* = 0.597) (Figure 3a,b). The values of BMD Mean and Low_5_ were significantly reduced at most regions after the functional loading on the implant (*p* < 0.04), except the AL and AC regions (*p* > 0.101). Those values decreased for the right-side regions of the condyle (*p* < 0.063), except the AL, AC, and PM regions (*p* > 0.101), and those values for the left side of the condyle were not different (*p* > 0.08) (Table 2 and Table 3).

## 4. Discussion

The methodology for temporomandibular joint osteoarthritis (TMJ OA) counts was developed to classify the clinical diagnosis of the TMJ condyle using the destructive change index (DCI), based on clinical CBCT images [21]. The number of subregions in the condyle was counted when TMJ OA-related bony changes were present. In the current study, the DCI was modified to quantify the morphological changes in the TMJ condyle using CBCT images from patients. A greater number of bony changes in the condyle were observed after implantation. Flattening was the most frequently observed change in both the right and left condyles before and after dental implantation, compared with erosion, osteophyte formation, sclerosis, and subchondral cyst formation. This result is consistent with the findings reported in other studies. Otterson et al. found that, in a group of nearly 160 older adults, the most common radiographic finding of the condyles on CBCT imaging was flattening, followed by osteophyte formation [27]. Similarly, the current study identified osteophyte formation as the second most prevalent change after condylar flattening.

Among the nine regional areas of the condyles, the three anterior regions (AM, AC, and AL) showed the highest prevalence of TMJ OA counts. These results are supported by the fact that the anterior region of the condyle is most susceptible to alteration during TMJ function, as it plays a critical role in rotation of the condylar head. For example, anterior disc displacement (ADD) is a common form of temporomandibular disorder (TMD) in which the articular disc is displaced toward the anterior surface of the mandibular condyle. ADD is likely to predispose the mandibular condyle to degenerative osseous changes [28].

Loss of teeth generally results in a decrease in biting force and perceived masticatory efficiency, but it may also be associated with effects extending beyond the oral cavity [29]. When missing teeth are restored with dental implants, biting forces return to near-normal levels or may even exceed normal levels [30]. This alteration in loading experienced by the bony structures of the TMJ may help explain the observed increase in the TMJ OA counts in these patients.

Because bone modeling and remodeling contribute to morphological changes, the distribution of BMD also changes. CT attenuation values (gray levels) were converted to BMD using the strong positive relationship with hydroxyapatite phantom densities [23,27,28,31,32]. The alteration in the BMD distribution results from the resorption of more mineralized pre-existing bone tissue followed by the addition of less mineralized newly formed bone tissue. Accordingly, the decrease in Low_5_ values reflects reduced mineralization associated with active new bone formation, which also lowers the mean BMD value. The frequency plot clearly demonstrated a shift of BMD toward lower values after implantation (Figure 1).

BMD values were higher in the anterior region than in other regions, corresponding with higher TMJ OA counts. These findings suggest that the anterior region of the condyle is dominantly loaded and adapts to increased loading by increasing BMD relative to other regions. Functional loading following crown placement on dental implants did not significantly change BMD in the anterior regions, whereas BMD decreased in the other regions. The reduction in BMD likely occurred because less mineralized newly formed bone tissue was added in those regions, as reflected by decreased Low_5_ values. These results suggest that functional loading after implantation increases loading on the mandibular condyle, triggering bone formation in regions with lower baseline BMD. This mechanobiological adaptation of condylar bone may help maintain load balance during mastication.

The mean BMD of the right condyle significantly decreased from before to after implantation. Interestingly, the right condyle initially exhibited a lower overall mean BMD and subsequently experienced a significant decrease during the observation period. In contrast, the left condyle did not show a significant change in the overall mean BMD. These findings support the results from Kim et al., who also reported significantly lower mean BMD and Low_5_ values in the right condyle compared with the left [22], although the underlying reason remains unclear. Increased masticatory function and/or other oral habits may contribute to enhanced bone remodeling on the right side. A strong correlation exists between preferred chewing side and the side with the most efficient chewing; however, previous studies have not demonstrated a consistent preference for chewing on one side [23,27,28,31,32,33]. It is also possible that implant location influenced the increase in right-sided bone remodeling, although the present study found that regions with lower BMD values were generally more susceptible to change.

Many previous studies indicate that mastication transmits greater loads directly to the bone surrounding a dental implant than to natural teeth, which are supported by a periodontal ligament that helps dissipate masticatory forces [4,5,6]. Natural teeth are capable of vertical movement of approximately 25–100 μm and buccolingual movement of 56–108 μm due to the surrounding periodontium, whereas dental implants exhibit only 3–5 μm of vertical movement and 10–50 μm of buccolingual movement [34]. Without periodontal ligament-supported occlusion to dissipate occlusal forces and provide proprioceptive feedback, patients with implant-supported occlusion may be more susceptible to occlusal overload and parafunctional activity [35]. Consequently, TMJ loading may increase following implantation.

Altered articulatory loading continuously stimulates bone modeling and remodeling, resulting in quantitative and qualitative changes in local subchondral bone [36,37,38]. These changes are regulated by osteoclast-mediated resorption and osteoblast-mediated bone formation. The CBCT-based BMD changes observed in this study reflect these biological processes. Because pre-existing bone tissue exhibits higher mineralization than newly formed bone tissue, skeletal adaptation produces a heterogeneous BMD distribution. Therefore, BMD distribution may serve as a surrogate marker for underlying mineralization dynamics [36,39]. Collectively, this study provides evidence-based proof of principle for the influence of dental implantation on the human mandibular condyle and offers baseline clinical data relevant to implant-related skeletal adaptation.

A limitation of the current study is the limited sample size. Among CBCT images obtained from 556 patients, fewer than 2% underwent longitudinal scans both at the time of crown placement on a single-tooth implant and after the functional loading period. Nevertheless, the results demonstrate the significant effects of implantation on the mandibular condyles. Another limitation is that the assessment of mandibular morphology was not feasible, as occlusal relationships were not standardized to centric relation or maximum intercuspation during CBCT image acquisition, resulting in variable degrees of mandibular opening. Growth patterns and facial types, such as mandibular divergence, may provide additional insight into the influence of biting forces and skeletal architecture on condylar BMD. Consequently, spatial relationships between the mandible and other skeletal structures, such as the maxilla or cranial base, could not be adequately evaluated.

## 5. Conclusions

In conclusion, the current findings show that dental implantation increases TMJ articulating loads during mastication, stimulating new bone formation to balance the load distribution on the mandibular condyle. To our best knowledge, this is the first study to demonstrate the effects of dental implantation on bony change in human TMJ condyles.

## Figures and Tables

**Table 1:** Comparisons of temporomandibular joint osteoarthritis (TMJ OA) counts and bone mineral density (BMD) parameters for the mandibular condyle between the left and right sides, and the time of crown placement on the implant (before) and after the functional loading period following crown placement on the implant (after). Significant (*p* < 0.05) or marginal (*p* < 0.07) differences are highlighted in **bold**.

Region	Side	Before	After	*p* Value
TMJOA Counts	**Both**	**3.73 ± 1.32**	**4 ± 1.41**	**0.058**
	Left	3.91 ± 1.22	4.18 ± 1.08	0.082
	Right	3.55 ± 1.44	3.82 ± 1.53	0.277
	*p* value (Left vs. Right)	0.42	0.531	
BMD Mean (mgHA/cm^3^)	**Both**	**1407.07 ± 118.39**	**1373.98 ± 116.33**	**0.024**
	Left	1424.89 ± 136.42	1394.65 ± 134.79	0.204
	**Right**	**1389.26 ± 100.63**	**1353.3 ± 130.71**	**0.058**
	*p* value (Left vs. Right)	**0.04**	0.133	
BMD SD (mgHA/cm^3^)	Both	181.28 ± 42.9	185.13 ± 45.46	0.416
	Left	179.84 ± 35.03	181.02 ± 44.71	0.888
	Right	182.72 ± 51.32	189.24 ± 53.54	0.205
	*p* value (Left vs. Right)	0.747	0.447	
BMD Low_5_ (mgHA/cm^3^)	**Both**	**1144.82 ± 86.24**	**1113.05 ± 86.1**	**0.004**
	Left	1169.18 ± 90.52	1144.82 ± 85.36	0.112
	**Right**	**1120.45 ± 78.22**	**1081.27 ± 84.02**	**0.02**
	*p* value (Left vs. Right)	**0.034**	**0.016**	
BMD High_5_ (mgHA/cm^3^)	Both	1727.5 ± 163.45	1703.05 ± 168.48	0.261
	Left	1746.55 ± 175.28	1717.82 ± 201.2	0.46
	Right	1708.45 ± 156.79	1688.27 ± 197.15	0.381
	*p* value (Left vs. Right)	**0.068**	0.523	

**Table 2:** Comparisons of regional BMD Mean (mgHA/cm^3^) between the time of crown placement on the implant (before) and after the functional loading period following crown placement on the implant (after). Significant (*p* < 0.05) or marginal (*p* < 0.07) differences are shown.

Region	Side	Before	After	*p* Value
AM	Right	1400.78 ±118.69	1359.27 ± 124.59	0.021
MM	Right	1337.39 ± 113.20	1289.61 ± 88.02	0.063
MC	Right	1359.13 ± 131.92	1306.34 ± 125.89	0.041
ML	Right	1393.69 ± 194.86	1351.09 ± 206.08	0.063
PC	Right	1362.78 ± 86.28	1288.03 ± 97.52	0.005
PL	Right	1344.16 ± 143.42	1281.96 ± 151.71	0.034

**Table 3:** Comparisons of regional BMD Low_5_ (mgHA/cm^3^) between the time of crown placement on the implant (before) and after the functional loading period following crown placement on the implant (after). Significant (*p* < 0.05) differences are shown.

Region	Side	Before	After	*p* Value
AM	Right	1151.82 ± 91.68	1111.91 ± 117.46	0.023
MM	Right	1102.27 ± 103.05	1059.27 ± 95.64	0.022
MC	Right	1127.27 ± 111.02	1066.09 ± 94.66	0.008
ML	Right	1145.55 ± 113.50	1097.18 ± 151.08	0.044
PC	Right	1139.91 ± 89.45	1065.27 ± 94.16	0.005
PL	Right	1148.82 ± 104.76	1082.30 ± 119.67	0.014

## Data Availability

The original contributions presented in the study are included in the article; further inquiries can be directed to the corresponding author.
